# mNGS-identified cellulitis due to quinolone-resistant *Edwardsiella tarda*: a case report

**DOI:** 10.3389/fmed.2024.1413561

**Published:** 2024-10-16

**Authors:** Xuejin Wang, Danxia Gu, Liwei Zhang, Yuchen Wu, Rong Zhang, Kewei Li, Haitao Ren

**Affiliations:** ^1^Department of Clinical Laboratory, The Second Affiliated Hospital of Zhejiang University, School of Medicine, Hangzhou, China; ^2^Key Laboratory of Medical Genetics of Zhejiang Province, Key Laboratory of Laboratory Medicine, Ministry of Education, School of Laboratory Medicine and Life Sciences, Wenzhou Medical University, Wenzhou, China; ^3^Laboratory Medicine Center, Department of Clinical Laboratory, Zhejiang Provincial People's Hospital (Affiliated People's Hospital), Hangzhou Medical College, Hangzhou, Zhejiang, China; ^4^Department of Vascular Surgery, The Second Affiliated Hospital, School of Medicine, Zhejiang University, Hangzhou, Zhejiang, China

**Keywords:** *Edwardsiella tarda*, cellulitis, virulence, resistant, metagenomic next-generation sequencing (mNGS)

## Abstract

*Edwardsiella tarda* is frequently isolated from aquatic animals and environments. While human infections caused by *E. tarda* are rare, some extraintestinal infections can be severe. This case report describes a patient with cellulitis of the right upper extremity of unknown origin. Metagenomic next-generation sequencing (mNGS) indicated that the patient was infected with *E. tarda*. Antimicrobial susceptibility testing revealed that the isolate was resistant to quinolones and trimethoprim/sulfamethoxazole. The isolate, positive for four virulence genes (*fimA, gadB, mukF*, and *sodB*), was confirmed to be virulent using the *Galleria mellonella* larvae model. Following early pus drainage and a 9-day course of imipenem, the patient ultimately recovered. This case report aimed to illustrate the presentation, diagnosis, and management of uncommon cellulitis caused by drug-resistant, virulent *E. tarda*.

## Introduction

*Edwardsiella tarda*, a Gram-negative intracellular bacillus within the Enterobacteriaceae family, was first identified by Ewing et al. (1). *E. tarda* is constantly detected in aquatic environments and aquatic animals, including fish, reptiles, and amphibians (2). Notorious as a pathogen causing edwardsiellosis in fish, which often leads to significant economic losses, *E. tarda* can also infect humans (3). Approximately 80% of human infections are gastrointestinal (2, 4) and tend to be self-limited. The remaining 20% of cases are extraintestinal infections, including bacteremia, wound infections, liver abscesses, cholecystitis, meningitis, and peritonitis. Previous reports have indicated that *E. tarda* can cause gastroenteritis in humans through the consumption of contaminated water or seafood, subsequently leading to bacteremia (5, 6). Predisposing factors, such as chronic liver cirrhosis and compromised immune function, increase susceptibility to *E. tarda* infections in humans (5, 7). In addition, *E. tarda* can cause extraintestinal infections, such as bacteremia (7), biliary tract infections (8), pneumonia (9), necrotizing fasciitis (10), peritonitis (11), and iliac psoas or epidural abscesses (12) (Table 1). These extraintestinal infections have been increasingly reported in recent years (6, 7, 13–15), with a notable mortality rate of 22.7% for severe cases involving bacteremia (16). These findings underscore the importance of a rapid diagnosis and appropriate treatment. In this study, we present a case of cellulitis caused by *E. tarda*, which was confirmed by metagenomic next-generation sequencing (mNGS). The use of advanced sequencing technologies such as mNGS has proven critical in the accurate and timely diagnosis of infections caused by rare pathogens.

**Table 1:** Extraintestinal cases of infections due to *Edwardsiella tarda* in humans.

**Year**	**Country**	**Age**	**Exposure history**	**Cause of diseases**	**Diseases history**
2016	Brazil	27	Episode of freshwater consumption upon drowning	Pneumonia, Bacteremia	–
2018	Japan	80	–	Cholangitis, Bacteremia	Diabetes
2018	Japan	65	Consumption of raw fish	Psoas and epidural abscess, Bacteremia	Gastric cancer
2019	Japan	64	–	Fasciitis, Bacteremia	–
2022	Thai	80	Fishmonger coming in contact	Peritonitis	Diabetes
2023	USA	20	Consumption of raw fish	Bacteremia	Long-term use of immunosuppressants

Cellulitis typically presents as an acute, spreading erythematous area with poorly demarcated borders, exhibiting the cardinal signs of inflammation, such as pain, fever, redness, and swelling. A systematic retrospective study in the United States found that cellulitis in immunocompetent adults is primarily caused by group A *Streptococcus*, with *Staphylococcus aureus* being a less frequent pathogen. The significant presence of Gram-negative bacteria might be attributed to patients with compromised immune systems, cirrhosis, aquatic injury exposure, or animal bite injuries (17). In the current study, we established the bacterial diagnosis by aspirating and culturing the patient's exudate, followed by rapid detection using metagenomic next-generation sequencing (mNGS), which confirmed an *Edwardsiella tarda* infection.

## Case description

A 60-year-old female patient was admitted to the Department of Burns and Wound Center, the Second Affiliated Hospital of Zhejiang University School of Medicine on 7 December 2023. The patient, without any evident traumatic cause, was experiencing continuous pain in the right thumb for 4 days, accompanied by edema in the last 2 days. Initially, the patient was treated with intravenous penicillin at a local hospital, but the condition did not improve. Then, localized congestion and necrosis of the thumb were observed, followed by abscess formation and restriction of movement. The patient visited our hospital for medical support and was diagnosed with cellulitis of the right upper extremity. She had no history of immune system disorders but did have chronic hypertension. The patient had no particular travel history to areas known for unique pathogens. The patient informed that she works as a seafood saleswoman with prolonged exposure to freshwater fish.

Upon physical examination, the patient was in good general condition, with no signs of pain or distress in her facial expression. Her breath sounds were clear upon auscultation, with no indication of shortness of breath. However, the skin on the right hand appeared red and swollen. There were localized areas of bruising and necrosis on the right hand, accompanied by limited mobility (Supplementary Figure S1).

### Diagnostic assessment and therapeutic intervention

Upon admission (Day 1), the blood tests revealed an elevated leukocyte count of 12.3 × 10^9^/L, a neutrophil count of 10.08 × 10^9^/L, an ultrasensitive C-reactive protein (CRP) level of 98 mg/L, and an interleukin-6 level of 12.27 pg/ml. The patient experienced pain, redness, and swelling localized to the palm of the right hand, particularly around the thumb where an abscess had developed. However, there was no accompanying fever. Since the patient was a seafood retailer with a history of seafood contact, Vibrio infection could not be ruled out. As for infections caused by *Vibrio vulnificus*, wound infection and primary septicemia are the most common manifestations. Wound infection might lead to necrotizing fasciitis, a severe infection of soft tissue and fascia. The skin could exhibit signs of fever, redness, swelling, ulcers, blisters, or black spots, and patients might experience intense pain, fever, chills, fatigue, diarrhea, vomiting, or purulent discharge from the infected area. In addition, cellulitis caused by *S. aureus* or *Streptococcus* could not be ruled out. Due to clinical suspicion of a bacterial etiology, the patient was started on empirical antibiotic therapy with imipenem (0.5 g every 8 h, intravenously) and amikacin (0.2 g daily, intravenously). Despite this treatment, the patient's response was found to be suboptimal.

On Day 2, to confirm the etiological diagnosis, we performed wound aspiration as part of the initial assessment to identify the bacterial pathogen. The aspirated fluid was submitted for microbiological culture and metagenomic next-generation sequencing (mNGS) analysis. The patient's wounds were carefully dressed and managed with regular changes every 3 days. On Day 3, the mNGS analysis detected the presence of *E. tarda* in the drainage fluid, with a read count of 9,291.

On Day 4, the clinical microbiological laboratory staff reported positive results for *E. tarda* in the drainage fluid. Antimicrobial susceptibility testing suggested that *E. tarda* was susceptible to cephalosporin and carbapenem, while resistant to quinolones and sulfamethoxazole (Table 2). Thus, the prescription of imipenem (0.5 g Q8H) continued for 8 days.

**Table 2:** Antimicrobial susceptibility testing of the *Edwardsiella tarda* isolate from the wound exudate.

**Antibiotics**	**MIC (μg/ml)**	**Zone diameter**	**Interpretation**
Piperacillin/tazobactam	≤ 1	–	Susceptible
Cefoperazone/sulbactam	≤ 8	–	Susceptible
Ceftriaxone	≤ 0.25	–	Susceptible
Cefepime	≤ 0.12	–	Susceptible
Cefuroxime	≤ 1	–	Susceptible
Meropenem	–	30	Susceptible
Ertapenem	≤ 0.12	–	Susceptible
Levofloxacin	≥8	–	Resistance
Trimethoprim/sulfamethoxazole	≥16/3	–	Resistance
Amoxicillin/clavulanic acid	16	–	Intermediate
Cefuroxime	≤ 1	–	Susceptible
Ceftazidime	≤ 0.12	–	Susceptible
Cefoxitin	≤ 4	–	Susceptible
Aztreonam	–	34	Susceptible
Imipenem	≤ 0.25	–	Susceptible
Amikacin	4	–	Susceptible
Ciprofloxacin	–	9	Resistance
Tigecycline	2	–	Susceptible

**Figure 1:**
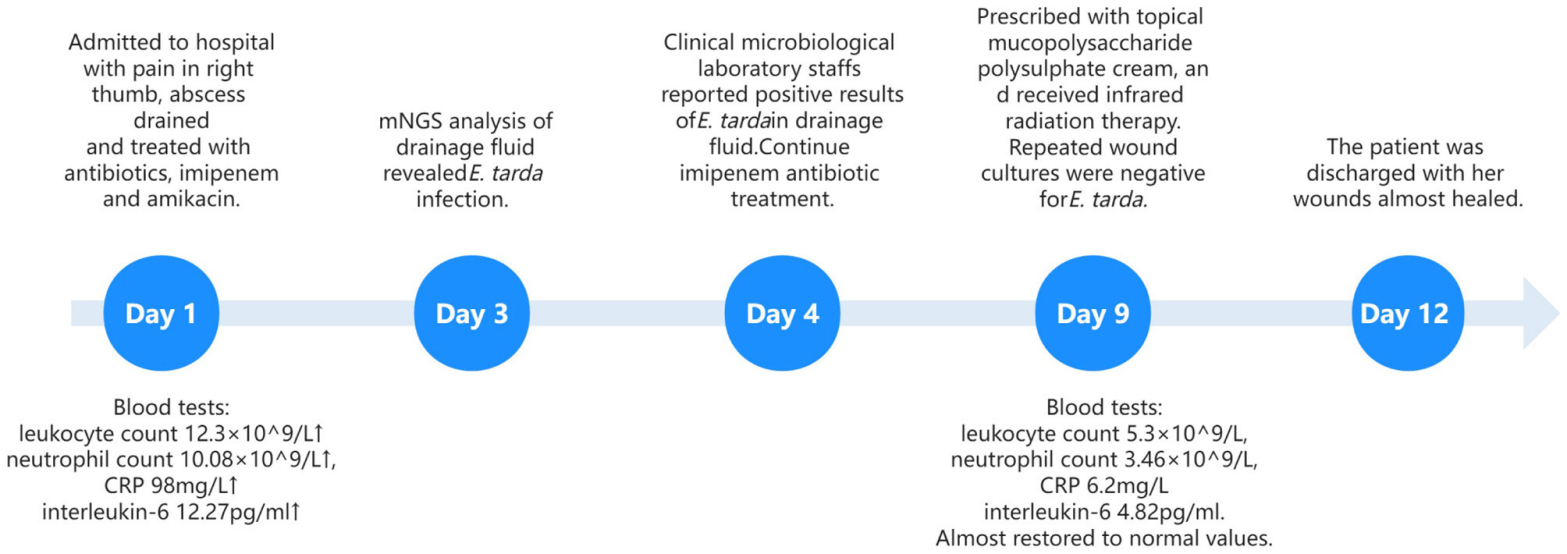
Treatment timeline of the patient.

On Day 9, the patient was prescribed topical mucopolysaccharide polysulfate cream and underwent infrared radiation therapy. The wound showed gradual signs of healing, and subsequent cultures of the wound swabs consistently yielded negative results. The inflammatory markers mostly returned to the normal values (Figure 1). The patient was discharged on Day 12. A schematic of the patient's treatment course is provided in Figure 1.

### Characteristics of *E. tarda* from the drainage fluid

#### Phenotypic characterization

The purulent exudate was streaked onto Columbia Blood Agar plates (Autobio, Zhengzhou, China) and incubated anaerobically at 37°C for hours. The isolates were confirmed as *E. tarda* using matrix-assisted laser desorption/ionization–time-of-flight mass spectrometry (MALDI-TOF MS), a technology provided by Bruker Daltonik GmbH (Bremen, Germany), known for its high-resolution microbiological identification capabilities.

Colonies from the culture plate were isolated and resuspended in 0.45% sterile saline to prepare a 0.5 McFarland standard bacterial suspension. A 145 μl aliquot was further diluted with 3 ml of the 0.45% sterile saline. The VITEK 2 Gram-negative AST card was selected, and antibiotic susceptibility testing was performed using the VITEK 2 Compact system (bioMérieux, France). Disk diffusion was also performed for meropenem, aztreonam, and ciprofloxacin. The results were interpreted following the guidelines in the M100 document from the Clinical and Laboratory Standards Institute (34th Edition).

**Figure 2:**
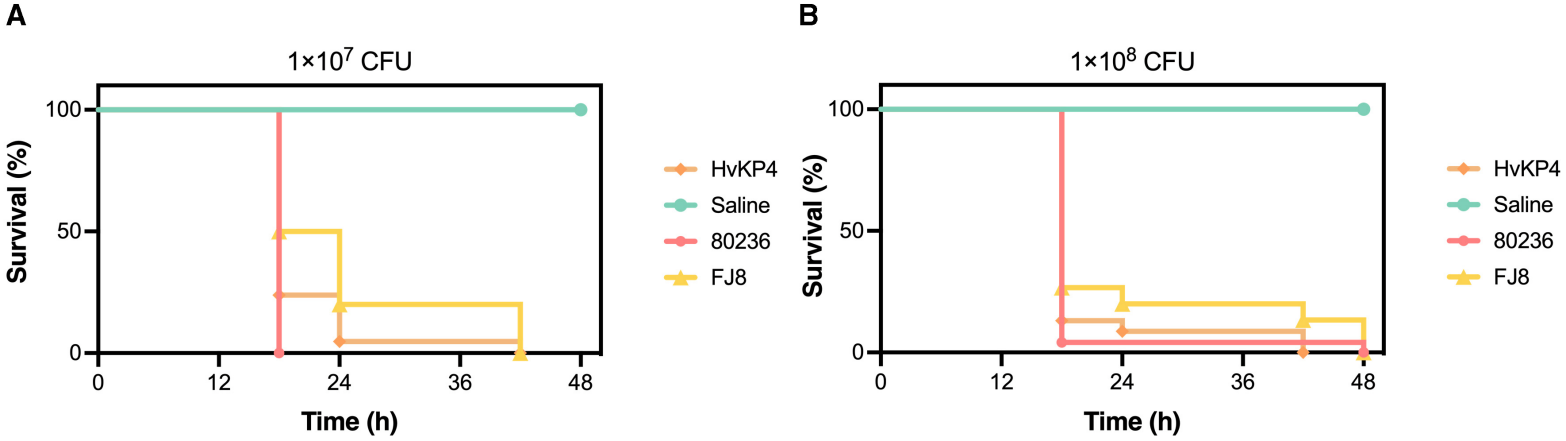
The virulence evaluation of *Edwardsiella tarda* 80236. **(A)** Survival graph of the wax moth larvae that were challenged by a dose of the *E. tarda* strain 80236 (10^8^ CFU/ml). **(B)** Survival graph of the wax moth larvae that were challenged by a dose of the *E. tarda* strain 80236 (10^7^ CFU/ml). The graphs are representative of the three independent experiments.

An *E. tarda* strain (named 80236) was isolated from the drainage fluid, and an assessment of its virulence was performed using the *Galleria mellonella* larvae model. Overnight cultures of *E. tarda* 80236 were adjusted with saline to concentrations of 1 × 10^7^ CFU/ml and 1 × 10^8^ CFU/ml. A 10 μl of the bacterial suspension was injected into each larva. The larvae were randomly divided into groups of eight larvae each and incubated at 37°C for 48 h. The survival rate of the larvae was recorded at 18, 24, 42, and 48 h after the injection. HvKP4, an ST11 hypervirulent *K. pneumoniae* strain, and FJ8, a *K. pneumoniae* strain of low-virulence, were used as hypervirulent and low-virulent controls (18), respectively. We analyzed the data using one-way ANOVA and Tukey's *post-hoc* test on GraphPad Prism version 9.0. Then, we found that the 1 × 10^7^ CFU/ml *E.tarda* concentration resulted in reduced larval survival, with almost 100% of the larvae dying within 18 h, as well as the *E. tarda* concentration of 1 × 10^8^ CFU/ml (Figure 2). Notably, *E. tarda* 80236 was more virulent than ST11 *K. pneumoniae* HvKP4 and FJ8.

#### Genomic characterization

For the mNGS analysis, we followed the protocol provided by BGI Genomics Co., Ltd., which included nucleic acid extraction, enzymatic digestion, DNA library construction, and circularization amplification. Subsequently, the sequencing was conducted using the MGISEQ-2000 genetic sequencer with the respective universal sequencing reagent kit, employing the probe-anchored polymerization sequencing method. After ~16 h of sequencing, the data were analyzed and compared using PMseq, infection pathogen nucleic acid detection software (19).

To further characterize the *E. tarda* isolate, the virulence genes and drug resistance genes were analyzed. We sequenced genomes using the NextSeq 500 sequencing platform (Illumina, San Diego, CA, USA). We trimmed or filtered raw reads to remove low-quality sequences and adaptors and assembled them *de novo* with the SPAdes Genome Assembler version 3.11.1 (20). Given the clinical importance of Antimicrobial Resistance (AMR) and the virulence of *E. tarda*, a targeted analysis of the acquired AMR genes and virulence-factor-associated genes was performed using ABRicate (https://github.com/tseemann/abricate) against the ResFinderFG v2.0 (21) database, PlasmidFinder (22) database, and the virulence factor database (VFDB) (http://www.mgc.ac.cn/VFs/; >90% identity and >75% coverage) (23). The genome assemblies of *E. tarda* 80236 have been deposited in the National Center for Biotechnology information (NCBI) and are registered under BioProject accession no. PRJNA1153709. All data are available from the corresponding authors upon reasonable request.

The presence of seven virulence genes, which include those associated with invasion (*fimA* and *esrB*), survival (*katB, sodB, citC*, and *gadB*), and proliferation (*mukF*), was assessed using BLAST analysis (https://blast.ncbi.nlm.nih.gov/Blast.cgi). Based on relevant reviews (24), the *fimA*-encoded fimbrial protein plays a critical role in the adherence of *E. tarda* to the host's tissues, facilitating bacterial colonization and the initiation of infection. The *esrB* gene controls the type III secretion system (T3SS), which is vital for the injection of effector proteins into the host's cells. This mechanism enables the bacteria to manipulate the host's cellular functions, promoting infection and evading the immune responses. In addition, the *katB* gene encodes catalase, which protects the bacteria from oxidative stress by neutralizing the reactive oxygen species produced by the host. Furthermore, the *gadB* gene, encoding glutamate decarboxylase, helps the bacteria survive in acidic environments, such as the host's gastrointestinal tract, during infection. In summary, these genes collectively contribute to *E. tarda's* ability to adhere, invade, and survive within the host, playing key roles in its pathogenicity and immune evasion.

## Discussion and conclusion

Humans are rarely infected by *E. tarda*, given its primary status as a fish pathogen. However, exposure to aquatic environments or consumption of improperly cooked aquatic animals remains the primary cause of infection for humans (2). *E. tarda* typically induces gastrointestinal inflammation; while extra-intestinal infections are uncommon, and they may result in potentially life-threatening conditions, with mortality rates reaching up to 50% (6).

Aquatic injuries, exposure to infected animals, certain dietary habits, and chronic underlying diseases are established risk factors for *E. tarda* infections (12). Soft tissue infections caused by *E. tarda* can facilitate the bacterium's entry into the bloodstream, especially in immunocompromised patients. Such infections can rapidly progress to life-threatening systemic sepsis. In severe cases, this poses a critical risk. A case report documented an instance of rare *E. tarda*-induced sepsis resulting from fishbone injury cellulitis in an Indian patient with an underlying hematological malignancy (25). According to a previous review, patients with soft tissue infections who developed bacteremia faced a significantly higher mortality rate, which reached 61.1% (6). In our case, the patient, a fishmonger by occupation, handles aquatic products on a daily basis. The cellulitis was likely caused by consistent contact with fish carrying *E. tarda*. Fortunately, an early diagnosis using metagenomic next-generation sequencing (mNGS) and prompt drainage of the abscess allowed for the timely administration of appropriate antibiotics, leading to a favorable patient outcome. Clearly, mNGS offers significant advantages in terms of timeliness and sensitivity, proving to be an invaluable diagnostic tool for identifying infections caused by rare and opportunistic pathogens.

*E. tarda* isolates are susceptible to most clinically administered antibiotics (26); however, they are resistant to benzylpenicillin, colistin, and polymyxin B (27). Empiric treatment options against *E. tarda* infections include beta-lactams, cephalosporins, aminoglycosides, and oxyquinolones (2). Alarmingly, multi-drug resistant *E. tarda* isolates are being increasingly reported among fishes (28, 29). However, there have been few reports of drug-resistant isolates in human infections. In 2011, Kawai et al. (4), reported the recovery of a trimethoprim/sulfamethoxazole-resistant *E. tarda* isolate from a pediatric patient in Japan with X-linked chronic granulomatous disease who was experiencing osteomyelitis. In this study, the *E. tarda* strain 80236 exhibited resistance to trimethoprim/sulfamethoxazole and quinolones, while showing intermediate susceptibility to amoxicillin-clavulanate. The emergence of drug-resistant *E. tarda* isolates in human infections warrants attention and raises concerns regarding antimicrobial resistance.

*E. tarda* has evolved through multiple mechanisms to cause infections in both humans and aquatic animals (30). Some studies revealed that the production of dermatotoxins and hemolysins, along with the ability to invade epithelial cells, resist phagocytosis, and evade serum-mediated killing, contributes to the pathogenesis of *E. tarda* (31). *E. tarda* 80236, possessing four virulence genes, demonstrated a significantly high level of virulence, as was confirmed by the *G. mellonella* larvae model. Notably, *E. tarda* 80236 was even more virulent than the hypervirulent *Klebsiella pneumoniae* isolate HVKP4 (18).

The hypervirulence of the isolate likely contributed to the rapid progression of cellulitis. Without the timely drainage and early administration of imipenem (0.5 g every 8 h intravenously), the patient's outcome might not have been as favorable. Clinicians should remain vigilant about the pathogenic potential of *E. tarda*. Metagenomic next-generation sequencing (mNGS) and microbiological identification are recommended for facilitating an early diagnosis. This should be swiftly followed by drainage and targeted antibiotic therapy to optimize favorable patient outcomes.

In summary, this report detailed a case of cellulitis caused by a quinolone-resistant and virulent strain of *E. tarda*. The patient was successfully managed with a combination of surgical drainage and antibiotic therapy. To mitigate the risk of *E. tarda* infection, it is advisable to avoid exposing wounds to aquatic environments and to ensure the consumption of only thoroughly cooked fish. Advanced microbiological techniques such as mNGS facilitate the early detection of pathogens, thereby enhancing clinical decision-making and treatment outcomes.

## Data Availability

The datasets presented in this study can be found in online repositories. The names of the repository/repositories and accession number(s) can be found in the article/Supplementary material.

## Supplementary material

Supplementary Figure S1 Localized bruising and necrosis presented in the right hand of the patient.
