# Supplementary figures and images for: mNGS-identified cellulitis due to quinolone-resistant Edwardsiella tarda: a case report
Source: Front Med (Lausanne). 2024 Oct 16;11:1413561. doi: 10.3389/fmed.2024.1413561 (PMC11521806; doi:10.3389/fmed.2024.1413561)

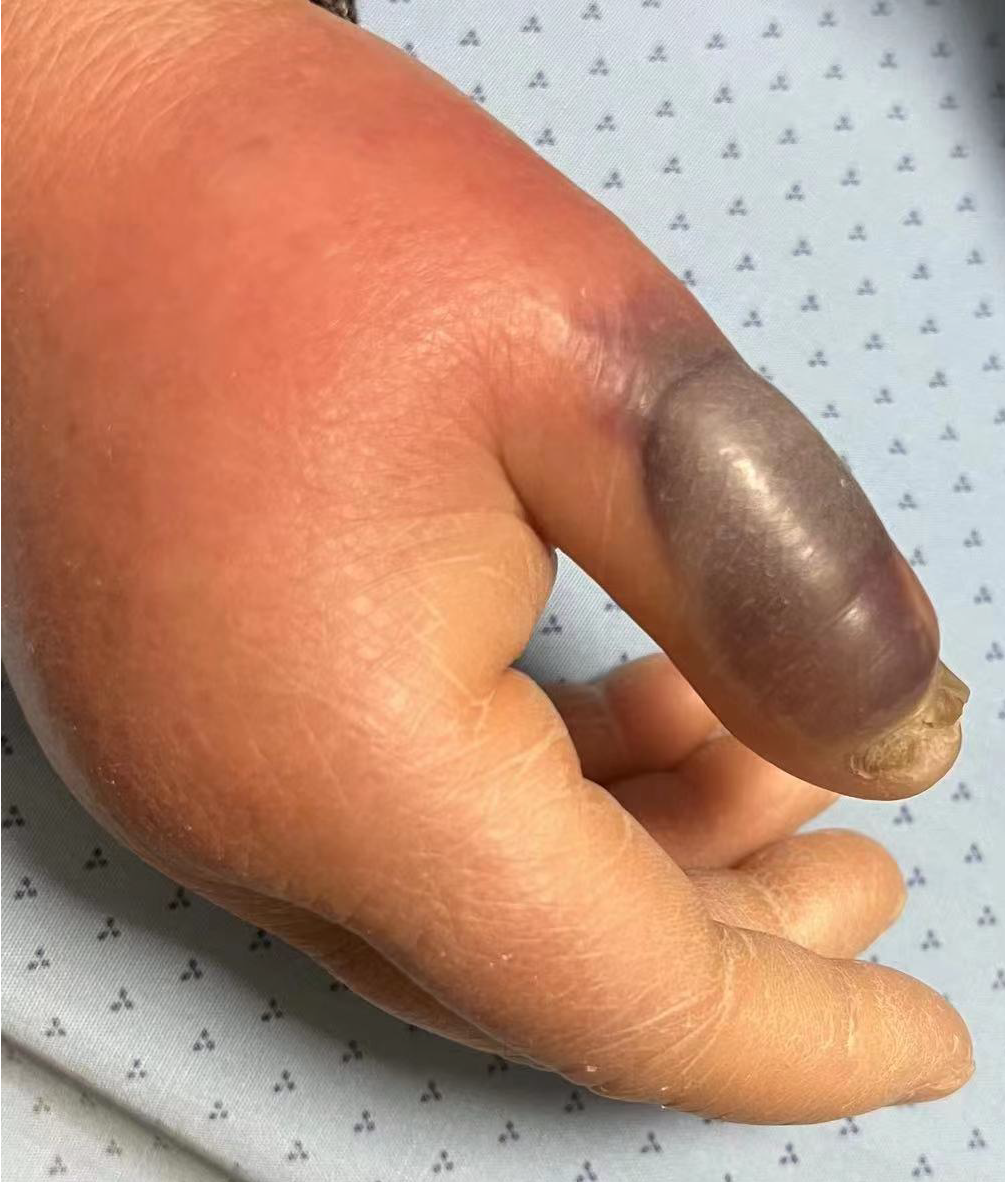

Supplement: Supplementary Figure S1 — Localized bruising and necrosis presented in the right hand of the patient. [file Image_1.tif]
